# Accuracy of Neutrophil-to-Lymphocyte Ratio in Predicting the Severity of Acute Appendicitis: A Single-Center Retrospective Study

**DOI:** 10.7759/cureus.45923

**Published:** 2023-09-25

**Authors:** Fahad S Al Amri, Raneem S Fihrah, Imtinan Al Jabbar, Rawan Alqahtani, Bayan Alnujaymi, Renad M Alshehri, Sarah S Asiri

**Affiliations:** 1 Surgery, Faculty of Medicine, King Khalid University, Abha, SAU; 2 College of Medicine, King Khalid University, Abha, SAU

**Keywords:** differentiation, complicated, appendicitis, acute, nlr

## Abstract

Objective: Investigate the potential use of the neutrophil-to-lymphocyte ratio (NLR) in the identification and differentiation of acute appendicitis patients, specifically between simple and complicated presentations.

Methods: An observational retrospective cohort study was conducted in Aseer Central Hospital, Saudi Arabia.

Results: In total, 103 patients were included in this study, in which males (50.5%) were more common than females (49.5%), and the most common age group was found to be 36-45 years (56.3%), followed by 18-25 years (23.3%). All patients (100%) had a diagnosis of acute appendicitis. The means of neutrophils, lymphocytes, and NLR were found to be 68.970%, 22.067%, and 5.020, respectively. The majority (69%) had non-complicated appendicitis, while (31%) had complications. A significant association was seen between NLR and the occurrence of complications (p-value = 0.00001).

Conclusion: This study can comprehensively support the evidence presented in the literature review. The use of the NLR demonstrates a notable level of accuracy in diagnosing acute appendicitis and differentiating between complex and uncomplicated cases.

## Introduction

Acute appendicitis is a prevalent etiology of abdominal pain across all age groups [1]. Nevertheless, despite the widely recognized classical symptoms and clinical characteristics associated with acute appendicitis, the early identification of this condition can occasionally present challenges due to the potential vagueness and uncertainty of its clinical manifestations [2, 3]. Failure to promptly diagnose acute appendicitis can lead to unfavorable consequences, such as perforation, which has the potential to cause substantial morbidity and mortality [3, 4].

Several scoring systems have been created to facilitate the diagnosis of acute appendicitis, including the Alvarado score [5] and the RIPASA score [6]. Nevertheless, the scoring systems now in use exhibit limited sensitivity and specificity, rendering them incapable of providing prognostic value in distinguishing between cases of simple and complex appendicitis [7]. In addition, it was found that an increased count of white blood cells (WBC) did not demonstrate more efficacy compared to the aforementioned grading systems in terms of predicting severity [8]. An increased level of bilirubin in the bloodstream has been identified as a potential indicator of appendix perforation [9, 10]. Nevertheless, it has been found that increased levels of C-reactive protein exhibit greater sensitivity in the detection of perforation in cases of acute appendicitis [11]. Currently, there is a lack of a conclusive instrument or indicator that can reliably forecast the diagnosis of acute appendicitis and effectively distinguish between uncomplicated and difficult cases with high levels of sensitivity and specificity. Therefore, the neutrophil-to-lymphocyte ratio (NLR) serves as a convenient and cost-effective indicator that may be derived from differential white blood cell (WBC) counts. The neutrophil count serves as an indicator of ongoing inflammation, whereas the lymphocyte count provides insight into the regulatory pathway [12].

The objective of this study was to elucidate the prognostic significance of the NLR in individuals presenting with symptoms of acute appendicitis as well as explore its potential to differentiate between cases of uncomplicated and severe appendicitis. Furthermore, the determination of cut-off values for the NLR in the context of appendicitis and complex appendicitis is of paramount importance.

## Materials and methods

Methodology

Study Design, Setting, and Population of Patients

This was an observational retrospective cohort study conducted to assess the neutrophil-to-lymphocyte ratio as a predictor to differentiate between complicated and non-complicated acute appendicitis at one of Aseer's hospitals; the cohort comprised patients who were admitted to the hospital with acute appendicitis. 

Inclusion and Exclusion Criteria

Adults with suspected acute appendicitis, emergency operations for acute appendicitis, and patients with an initial evaluation that included measurement of neutrophil and lymphocyte counts were included. Patients with autoimmune diseases, chronic infections, known cases of hypersensitivity, and pregnant ladies were excluded. 

Sampling Size and Technique and Data Collection, Management, and Analysis

103 patients were selected and included randomly using a simple random sampling technique. Patients who had been diagnosed with acute appendicitis at the hospital within the timeframe of 2018-2022 were enrolled in the study, and they were grouped according to age and gender via general demographic. Furthermore, physical file records of patients matching our inclusion criteria were obtained from the inpatient office after receiving IRB approval. Pre- and post-operative complete blood count test results, along with the final working diagnosis, were gathered and interpreted by the researchers. The data collected was computerized through Microsoft Excel. The data was analyzed using SPSS Version 27 (IBM Corp., Armonk, NY). The data was presented graphically (frequency tables, graphs).

Ethical considerations 

The research staff was thoroughly informed about this research regarding its purpose, method, and any possible risks involved. All data was kept in a secure place; the data was kept private with authorized access only. The research staff ensured confidentiality and privacy. Also, any private information was not discussed anywhere during the study's analysis. All types of formats containing data were disposed of properly after the completion of the study.

## Results

**Figure 1:**
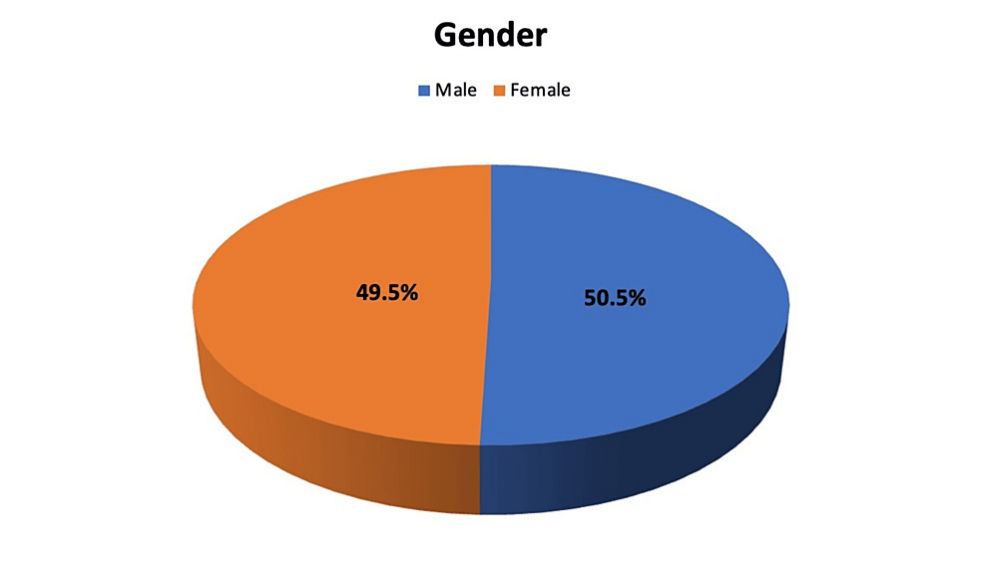
Showing gender distribution of patients.

As shown in Figure 1, a total of 103 patients were utilized in the investigation being analyzed in this research study. There were a total of 51 (49.5%) women and 52 (50.5%) men who participated in this study. The percentage of male participants was slightly higher than the percentage of female participants.

When we analyze the age distribution of the patients under investigation in more detail, the predominant age group among our study participants was 36-45 years, which accounted for an impressive 56.3% of the total cohort of 58 participants. Subsequently, the 18-25-year age group was shown to be the second-most prevalent, constituting 23.3% of the total sample population with 24 patients. Near behind was the 26-35-year age group, which comprised 21 patients and accounted for 20.4% of the overall distribution, as shown in Table 1.

**Table 1:** Showing the age distribution of patients.

Table 1					
		Frequency	Percent	Valid Percent	Cumulative Percent
Valid	18-25 years	24	23.3	23.3	23.3
	26-35 years	21	20.4	20.4	43.7
	36-45 years	58	56.3	56.3	100.0
Total		103	100.0	100.0	

Table 2 shows the hematological parameters of the patients that were analyzed in this study. The neutrophil percentage (mean: 68.970%, SD: 13.762), lymphocyte percentage (mean: 22.067%, SD: 12.598), and the NLR (mean: 5.020, SD: 4.899) mean values and standard deviations were calculated. These parameters shed light on the inflammatory and immune profiles of the population under study.

**Table 2:** Shows the hematological parameters of the patients in the study. NLR: neutrophil-to-lymphocyte ratio

Parameter	Mean	Standard deviations (SD)
Neutrophils %	68.970	13.762
Lymphocytes %	22.067	12.598
NLR	5.020	4.899

**Figure 2:**
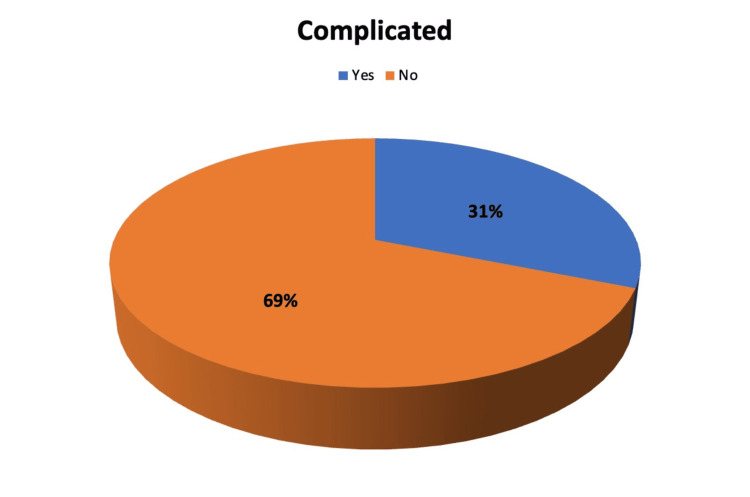
Showing if the acute appendicitis was complicated or non-complicated.

Figure 2 shows that out of the 103 patients, 71 (or 69%) experienced non-complicated acute appendicitis, while the remaining 32 (or 31%) experienced complicated acute appendicitis.

Table 3 shows a significant association, which was seen between NLR and the occurrence of complications (p-value=0.00001). The chi-square statistic is 32.4043. The p-value is < 0.00001 (significant at p < 0.05).

**Table 3:** Showing the correlation between NLR and the occurrence of complications. NLR: neutrophil-to-lymphocyte ratio

	Normal NLR (less than 3)	Abnormal NLR (3 or more)	Total
Complications	1	31	32
No complications	45	26	71
Total	46	57	103

## Discussion

The objective of this study was to investigate the potential use of the NLR in the identification and differentiation of acute appendicitis patients, specifically between simple and complicated presentations.

Various and different published studies were done about the same topic as the present article. In 2022, Sipra et al. conducted a study in Saudi Arabia. Children with acute appendicitis showed a higher NLR than healthy children. Acute appendicitis was ruled out at two cut-off values of NLR, with 94% sensitivity and 100% specificity. An accurate marker for acute appendicitis was a high NLR of 3.5. Significant differences in NLR values were also seen in the severity of appendicitis. The results of the previous study align with those found in the current study (shown in Table 2), in which the mean of NLR was high in both studies (3.5 in the previous study and 4.5 in the current study) as an accurate marker for acute appendicitis [13].

When comparing the results of the current study to the results of the previous study done by Ayeni et al., it can be noticed that a significant difference was found between the NLR and PLR ratios in the previous study (p = 0.05, Kruskal-Wallis). For uncomplicated appendicitis, the area under the ROC curve (AUC) and the cut-off for neutrophil-to-lymphocyte ratio and PLR were 0.741 and 3.80, respectively. In complicated appendicitis, using neutrophil-to-lymphocyte ratio and PLR, the AUC and cut-off were 0.776 and 8.86, respectively. Meanwhile, in the current study, the results agree with those found in the previous study (shown in Table 3), in which a significant association was seen between NLR and the occurrence of complications (p-value = 0.00001) [14].

The research conducted by Hajibandeh et al. in 2020 established a cut-off value of 4.7 for the neutrophil-to-lymphocyte ratio in diagnosing appendicitis. The sensitivity and specificity of this cut-off value were found to be 88.89% and 90.91%, respectively, with an AUC of 0.96. The cut-off value for complex appendicitis was determined to be 8.8 for the neutrophil-to-lymphocyte ratio. This number exhibited a sensitivity of 76.92% and a specificity of 100%, resulting in an AUC of 0.91. A neutrophil-to-lymphocyte ratio over 4.7 was found to be a significant indicator of acute appendicitis (odds ratio [OR]: 128, p < 0.0001). Furthermore, a neutrophil-to-lymphocyte ratio surpassing 8.8 was identified as a strong predictor of complicated appendicitis (OR: 43, p < 0.0001). Once again, the findings of this study are consistent with those of the recent study. However, it is worth noting that the previous study reported a higher NLR value (>8.8) related to problems compared to the current study, where the NLR value was found to be greater than 3 (as indicated in Table 3) [15].

In a study conducted by Ahmad et al., the authors reported the median values of the NLR for three distinct groups. Specifically, the median values for groups 1, 2, and 3 were found to be 2.37, 5.25, and 9.27, respectively. A statistically significant difference was seen in the NLR between group 1 and group 2 (P < 0.001), as well as between group 2 and group 3 (P < 0.001). The diagnostic values of the NLR for acute appendicitis and perforated appendicitis were determined to be 3.11 (with a sensitivity of 75.23% and specificity of 68.70%) and 6.17 (with a sensitivity of 76.32% and specificity of 58.72%), respectively. A significant association was seen between the NLR and the severity of the disease. Furthermore, a discernible association was observed between the NLR and the length of hospital stay. In the present investigation, the average values for neutrophils, lymphocytes, and NLR were determined to be 67.5%, 23.0%, and 4.5, respectively, as depicted in Table 2. Furthermore, a noteworthy correlation was observed between NLR and the incidence of complications, with a p-value of 0.00001 [16].

Lastly, in a study conducted by Prasetya et al., it was observed that the acute appendicitis group demonstrated a significant rise in both neutrophil count and NLR when compared to the control group. Nevertheless, no statistically significant disparity was detected in the WBC when comparing the two groups. Moreover, the study demonstrated a notable increase in the number of neutrophils and the neutrophil-to-lymphocyte ratio in instances of complex appendicitis when compared to cases of uncomplicated appendicitis. The diagnostic performance measures of the NLR for acute appendicitis were determined to be as follows: a level of sensitivity of 83.5%, specificity of 57.7%, positive predictive value (PPV) of 81.4%, negative predictive value (NPV) of 61.2%, area under the receiver operating characteristic (ROC) curve of 0.764, and a cutoff point of 2.87. However, it is crucial to take into account the sensitivity, specificity, PPV, NPV, and area under the ROC curve as significant measures. In the present investigation, the study findings revealed that the average percentages of neutrophils, lymphocytes, and NLR were determined to be 67.5%, 23.0%, and 4.5, respectively (see Table 2). Furthermore, a statistically significant correlation was observed between NLR and the occurrence of complications (p-value = 0.00001) (Table 3), which aligns with the outcomes of a prior study [17].

Recommendations

NLR, along with other laboratory parameters, should be used to predict and assess the outcomes of appendicitis. NLR should be used to reduce the rate of negative appendectomies. More studies should be conducted regarding this topic to support the evidence and improve the outcomes.

## Conclusions

Overall, the capacity to anticipate the likelihood of complications in cases of acute appendicitis has the potential to facilitate prompt intervention and reduce morbidity. The utilization of the neutrophil-to-lymphocyte ratio has been found to be a valuable and consistent tool in the diagnostic process of acute appendicitis; this study can support the evidence found in the literature review. The use of the NLR demonstrates a notable level of accuracy in diagnosing acute appendicitis and differentiating between complex and uncomplicated cases.
